# Intercultural Childbirth: Impact on the Maternal Health of the Ecuadorian Kichwa and Mestizo People of the Otavalo Region

**DOI:** 10.1055/s-0040-1721353

**Published:** 2021-01-29

**Authors:** Susana Eulalia Dueñas Matute, Edson Zangiacomi Martinez, Eduardo Antônio Donadi

**Affiliations:** 1 Department of Social Medicine, Ribeirão Preto Medical School, Universidade de São Paulo, Ribeirão Preto, SP, Brazil; 2 Facultad de Ciencias Médicas, Cátedra de Pediatría, Universidad Central del Ecuador, Quito, Ecuador; 3 Department of Internal Medicine, Ribeirão Preto Medical School, Universidade de São Paulo, Ribeirão Preto, Brazil

**Keywords:** maternal mortality, intercultural childbirth, Kichwa, Otavalo, Ecuador, mortalidade materna, parto intercultural, Kichwa, Otavalo, Equador

## Abstract

**Objective** Considering the increased frequency of maternal deaths reported from 2001 to 2005 for Indigenous and mestizo women from the Ecuadorian rural area of Otavalo, where the Kichwa people has lived for centuries, the objective of the present article is to describe how the efforts of the local health community and hospital workers together with a propitious political environment facilitated the implementation of intercultural childbirth, which is a strategy that respects the Andean childbirth worldview.

**Methods** We evaluated a 3-year follow-up (2014–16) of the maternal mortality and the childbirth features (4,213 deliveries).

**Results** Although the Western-style (lying down position) childbirth was adopted by 80.6% of the pregnant women, 19.4% of both mestizo and Indigenous women adopted the intercultural delivery (squatting and kneeling positions). Both intercultural (42.2%) and Western-style (57.8%) childbirths were similarly adopted by Kichwa women, whereas Western-style childbirth predominated among mestizo women (94.0%). After the implementation of the intercultural strategy in 2008, a dramatic decrease of maternal deaths has been observed until now in both rural and urban Otavalo regions.

**Conclusion** This scenario reveals that the intermingling of cultures and respect for childbirth traditions have decreased maternal mortality in this World Health Organization-awarded program.

## Resumo

**Objetivo** Considerando a crescente frequência de mortes maternas notificadas de 2001 a 2005 entre mulheres indígenas e mestiças da área rural equatoriana de Otavalo, onde o povo Kichwa vive há séculos, o objetivo deste artigo é descrever como os esforços da comunidade local de saúde e dos trabalhadores hospitalares, juntamente com um ambiente político propício, facilitaram a implementação do parto intercultural, que é uma estratégia que respeita a visão de mundo do parto andino.

**Métodos** Foram avaliadas as características da mortalidade materna e do parto (4.213 partos) por um período de 3 anos (2014–16)

**Resultados** Embora o parto no estilo ocidental (posição deitada) tenha sido adotado por 80,6% das gestantes, 19,4% das mestiças e indígenas adotaram o parto intercultural (posições de agachamento e ajoelhamento). Os partos interculturais (42,2%) e ocidentais (57,8%) foram adotados de maneira semelhante pelas mulheres Kichwa, enquanto o parto ocidental predominou entre as mestiças (94,0%). Após a implementação da estratégia intercultural em 2008, foi observada uma redução drástica de mortes maternas nas regiões rurais e urbanas de Otavalo.

**Conclusão** Esse cenário revela que a mistura de culturas e o respeito às tradições do parto diminuíram a mortalidade materna neste programa premiado pela Organização Mundial de Saúde.

## Introduction

Maternal mortality has been considered a serious and persistent challenge worldwide. In 2015, most of the 303,000 maternal deaths occurred in low-income countries, primarily due to inequities in the access to health services, underlined by large disparities between countries in rural and urban populations, and cultural differences. 1 2 Approximately 16 women die every day from pregnancy or childbirth in Latin America and in the Caribbean countries, with hemorrhages, infections and pregnancy-induced hypertension being the most common causes of maternal mortality. 3 4 In the years 2000, 2005, 2010, 2015, and 2017, the maternal mortality ratios (MMRs) in Ecuador were, respectively, 122, 94, 78, 63, and 59 deaths per 100,000 live births. 5 6 Maternal mortality ratio is a central indicator in the Sustainable Development Goals proposed by the United Nations, and efforts should be made to decrease this elevated number of deaths. 7

Indigenous peoples present poor health indicators throughout the world, characterized by inequality and inequity between Indigenous and non-Indigenous populations, which is the result of socioeconomic conditions due to the historical social exclusion of these peoples. 8 Ecuador has not been the exception, so only 30% of the Indigenous population has access to specialty care, contrasting with 80% of non-Indigenous women. 9 The Law for the Provision of Free Maternity and Child Care (in Spanish, *Ley de Maternidad Gratuita y Atención a la Infancia* , LMGAI) was an incremental health policy first introduced in 1994 and officially made into a mandate in 2002, as the result of the concerted effort of key stakeholders to improve health outcomes for the most vulnerable Ecuadorian women. 10 As a positive impact of the LMGAI, an increase of the number of institutional births by 39% was noted. 10 However, an ecological study using data for 2014 from the 24 Ecuadorian provinces showed that the MMR was higher in the provinces with the highest percentage of Indigenous population. 11

Despite improving demographic and economic disparities, Indigenous women in rural areas do not have access to formal health services. 12 A report from the United Nations (UN) Economic Commission for Latin America and the Caribbean (ECLAC), including disaggregated health care utilization according to the Indigenous status of women, showed that, in 2004, the percentage of Ecuadorian Indigenous women who attended prenatal care, gave birth in a health facility, and received follow-up attention was systematically lower than among non-Indigenous women. 13 14 In addition, from 2001 to 2005, an increased number of maternal deaths, primarily Indigenous women from the rural areas, were observed in the Ecuadorian area of Otavalo. 15

With the aim of improving maternal health conditions for Indigenous women and reducing maternal mortality, a pioneering project has been implemented at the San Luis de Otavalo Hospital, in Ecuador. This project aimed to encompass the knowledge, beliefs, and rituals of the pregnant Kichwa women in the health care. Considering that interculturality is the recognition of differences, paying respect to the values of each culture, 16 17 18 19 several strategies were included to reduce maternal death, primarily in the rural areas of Otavalo. In 2007, a sustained process of community and hospital health personnel training was initiated to introduce the intercultural childbirth. This strategy was able to reduce the Kichwa and mestizo maternal mortality in the following years, and this program was awarded by the World Health Organization (WHO) in the category “Good Practices and Safe Maternity.” 20 In the present study, we further evaluated the impact of intercultural childbirth on maternal mortality and the compliance of Kichwa and mestizo women to the cultural appropriate delivery (squatting, kneeling, and standing positions) in comparison to the Western-style (lying down position) delivery.

## Methods

### Setting

The present retrospective study evaluated all pregnant Indigenous and non-Indigenous women from the Otavalo canton who gave birth at the San Luis de Otavalo Hospital (SLOH), from 2014 to 2016. A total of 4,213 women was studied, of whom 1,560 (aged 13–45 years, median 33.5, predominating women aged 20–35 years) were Indigenous and 2,653 (median 34.5) were mestizo. Data regarding the total number of deliveries, number of intercultural deliveries and maternal mortality were retrieved from the database and statistical register of the SLOH, according to the proceedings of the Pan American Health Organization (PAHO) and of the Ecuadorian Ministry of Public Health. Since SLOH does not have a computerized information system, all data were obtained from non-electronic medical records. Written informed consent was not feasible because the present study was based on retrospective data.

### Intercultural Birth

The definition of intercultural childbirth adopted here includes, among other aspects, the birth of a baby respecting cultural traditions, in a comfortable position for the mother. According to the cultural values of the Kichwa woman, childbirth is performed adopting the vertical position (standing, sitting, squatting, kneeling, semi-sitting), while being attended by the midwife. 21 To perform intercultural childbirth, several actions were taken by the Otavalo hospital, including, i) the woman's right to choose the mode of delivery; ii) the implementation of a physical area according to the cultural Indigenous conception to delivery care, including a small kitchen to heat water to make herb infusions; iii) the identification, training, evaluation, and certification of the canton midwives to take care of the patients before and after childbirth, and permanent accompaniment of the patient during labor; iv) permission for the husband or another family member to stay in the room during labor and to cut the umbilical cord after delivery; v) the provision of adequate clothes to the mother, such as a sterilized flannel dress with a wide abdominal opening to keep the patient warm and to respect pregnant woman privacy; and vi) authorization and permission for traditional practices that are harmless to health. 21 According to the Kichwa traditions, these practices include the permission to use a coriander seeds herbal infusion to prevent pain during delivery and a fig leaf infusion to prevent bleeding after delivery; permission for the patient to have chicken broth before delivery and broth made of a young lamb's head after delivery; and the granting of the placenta to the family to bury it in their homes. 21

### Features of the Otavalo Canton

The 24 Ecuadorian provinces are divided into cantons, and these cantons are divided into parishes (the smallest administrative units). The Otavalo canton is located in the province of Imbabura, in the northern part of Ecuador, presenting an area of 579 km ^2^ . The city of Otavalo is the capital of the canton of the same name, situated 110 km from the capital Quito at a height of 2,565 m above sea level at the following coordinates: 78° 15' 49' West longitude and 0° 13' 43”' North latitude. Of the 145,000 inhabitants (51.9% woman), 62.3% are of the Kichwa nationality (Cayambis and Otavalos) and 37.5% are mestizos ( *Instituto Nacional de Estadística y Censos* , all information retrieved from www.ecuadorencifras.gob.ec ). The Otavalo canton has two urban and nine rural parishes. The economically active population is 52.3%, and the main productive activities are trade (82.5%), manufacturing industries (3.4%) and other professions (3.4%). Poverty reaches 32.8% of the population and illiteracy predominates among women (22.5%) compared with men (12.7%). The city is well known for its handicrafts and music, but economic development is uneven, rich Indigenous people tend to live in urban areas, but the vast majority of the Indigenous populations live in rural areas, where access to health services at childbirth is more difficult. Otavalo has a major primary care hospital (SLOH) for urban and rural populations and an ambulatory care center only for urban outpatient care.

## Results

Of the 4,213 births attended at the SLOH from 2014 to 2016, 19.4% were intercultural births and 80.6% were conventional childbirth. Considering childbirth among the Kichwa women, 42.2% adopted the intercultural childbirth, and 57.8% adopted the Western-style delivery. Among the non-Indigenous women, 6.0% preferred the intercultural childbirth and 94.0% preferred the conventional delivery. Maternal mortality has remained at 0/100,000 live births, maintaining the same scenario since 2008, the year in which the intercultural delivery strategy was implemented. Among all patients who preferred the intercultural childbirth, the squatting (49.7%) and kneeling (46.7%) positions were preferred when compared with the seating position (3.5%). Table 1 summarizes these results.

**Table 1:** Childbirth features and maternal mortality among rural and urban Indigenous and mestizo women of the Otavalo canton attended

	Year			Total	
	2014	2015	2016	n	%
Total deliveries	1,417	1,337	1,459	4,213	100.0
Intercultural childbirth	300	219	299	818	19.4
Western style childbirth	1,117	1,118	1,160	3,395	80.6
Deliveries among Kichwa	
Intercultural childbirth	261	198	200	659	42.2
Western style childbirth	94	118	689	901	57.8
Deliveries among mestizo	
Intercultural childbirth	39	21	99	159	6.0
Western style childbirth	1 023	1 000	471	2 494	94.0
Maternal mortality	0	0	0	0	0

## Discussion

Compared with other Latin American countries, the access to health services for the Indigenous population is greater in Ecuador than in Mexico and Peru. Beginning in 2008, after the successful experience in Otavalo, intercultural childbirth has been established as a public policy of the Ministry of Public Health, which helped to reduce the intercultural distance between the Indigenous and non-Indigenous populations.

A report published in 2017 by the Epidemiological Surveillance System of Ecuador register a striking decreased number of maternal deaths compared with the previous decades. 22 In 2001, the provinces that exhibited the highest number of general mortality were those in the Central and Northern areas (Chimborazo, Cotopaxi and Tungurahua), where rural Indigenous populations are predominant. In 2016, the provinces with the highest number of deaths were those in the coastal zone (Guayas, Manabí and Esmeraldas), while the Northern and Central provinces reported lower rates of maternal mortality. It is noteworthy that in the provinces of Chimborazo, Imbabura (Canton Otavalo), and Tungurahua, which are areas with a large Indigenous rural population and that historically exhibited the highest rate of maternal mortality, this rate has also dropped greatly. 22

International health organizations, such as the WHO, the International Federation of Gynecology and Obstetrics (FIGO), and the International Confederation of Midwives, have been establishing strategies and policies for the promotion of pregnancy, childbirth, and newborn care. Given the challenges of improving health care, comprehensive strategies such as the cultural childbirth have been proposed, including the needs of the pregnant women and their rituals and beliefs related to the pregnancy and childbirth processes. 23 These strategies aim to impart knowledge, respect indigenous cultures, and understand their specific needs and motivations to ensure better access to health services, thus breaking the cultural barriers that may exist between two ethnic populations that share the same territory. In this sense, the introduction of intercultural strategies in the public health policy has contributed to reduce maternal mortality, primarily in the rural Indigenous sectors by providing access to health services. Taking into account that Ecuador still maintains a high number of maternal deaths (43/100,000), 24 the striking reduction of the Indigenous and non-Indigenous maternal mortality in Otavalo is noteworthy. Different social and political actors contributed to the successful strategy of intercultural childbirth, including: i) the new Ecuadorian Constitution (2008) that widened the rights to indigenous people, recognizing the presence of indigenous leaders in strategic positions, ii) by the first time, an Indigenous hospital manager was appointed to take care of the Indigenous health care at the Otavalo canton, iii) the empowerment of the internal (technical and administrative staff of the hospital) and external actors (local government, non-governmental organizations, midwives, and the indigenous health facilities). 25 26 In this context, it is important to emphasize the role of Indigenous midwifes who played a pivotal linkage between the rural Indigenous communities and the hospital staff, and provided maternal care during the pre and postchildbirth periods. A group of 36 midwives were certified by the Ministry of Health to participate in the intercultural strategy; however, just one midwife, who is paid by the government, accompanies the women in the intercultural hospital facility. Although other midwifes are not paid by their services, they are proud of their roles on helping Kichwa women before and after delivery. In addition to these actors, private physicians who worked in Quito and had experience in performing vertical childbirth contributed to the success of the humanized childbirth.

Overall, despite the introduction of the humanized childbirth, the conventional Western-style childbirth continues to be the most frequent position, and it was adopted by 80.6% of the women, contrasting with 19.4% of women who adopted the intercultural childbirth. Notwithstanding, intercultural childbirth was primarily adopted by the Kichwa women and, at a lesser extent, by the mestizo women. It is worth mentioning that the squatting and kneeling positions were the chosen ones by Indigenous and non-Indigenous women. The vertical childbirth does not represent only a delivery position, but an integral attention to the women, respecting their beliefs, rituals, and Andean cosmovision. Considering that public health institutions have historically adopted the Western-style childbirth, the implementation the humanized childbirth represents a new approach to the acceptance of public health institutions to this different concept of childbirth attention. Since the introduction of the intercultural childbirth in Ecuador in 2007, this strategy has also been adopted in other Ecuadorian regions. 26 The Ministry of Heath determined that all provinces and cantons with major Indigenous populations must have two facilities for childbirth, including an intercultural one. Despite this public policy, that had encompassed all indigenous communities, this WHO awarded strategy provided to the Otavalo canton one of the best maternal survival rates in Ecuador.

Considering that the MMRs observed in Ecuador were 63 and 59 deaths per 100,000 live births in 2015 and 2017, respectively, a maternal mortality equal to zero reported in this period in SLOH highlights the importance of studies about intercultural childbirth. 5 However, since it would not be expected for mortality to be higher for women attended inside health facilities, although submitted to traditional practices, it is difficult to conclude that a possible reduction of the mortality in SLOH is necessarily due to intercultural childbirth. In contrast, Otavalo is not the area of the country with the highest MMR, and the pioneer practice of intercultural childbirth of SLOH provides a basis for new studies for better understanding of their benefits and how the intercultural health policy could be implemented. 25

Our study has important limitations. The SLOH does not have an electronic medical record system, and collection of data from medical charts had to be done manually. Consequently, information about maternal and neonatal outcomes, such as postpartum hemorrhage prevalence, Apgar scores, hospitalization time, and infections were not adequately available. Our results are based on mortality alone, and this limits our results. In addition, MMRs in the years preceding the adoption of the intercultural childbirth by the SLOH are also not available. As another limitation of the present study, the database was not adjusted for missing data as the WHO estimates are. Since we used retrospective data, important perceptions of the women about aspects of the intercultural births were not possible to obtain. Therefore, future prospective studies should be conducted to investigate intercultural births and their possible impacts on maternal health, including more adequate information regarding maternal and neonatal outcomes.

Despite all these problems, the present article is an important first step in exploring the possible impacts of intercultural childbirth adopted in SLOH on maternal mortality and serves as motivation for improvements in the hospital information system to enable more complete studies. San Luis de Otavalo Hospital was a pioneer institution in the implementation of “vertical birth” in Ecuador, as an intercultural health policy practice that aims to increase the access of Indigenous women to maternity care. 25 27 We can understand that this intercultural strategy alone was not the sole factor that strongly influenced the reduction of maternal mortality.

## Conclusion

However, communion between the rural Indigenous communities and the public institutions possessing an integral concept regarding childbirth attention has contributed to reduce maternal mortality among Indigenous populations. As a corollary to this strategy, an important hitchhiking effect was also observed for mestizo women, and further investigation is encouraged.

## References

[1] WHO, UNICEF, UNFPA, World Bank Group, United Nations Population Division Trends in maternal mortality: 1990 to 2015 [Internet] Geneva WHO 2015[cited 2020 Mar 9]. Available from:http://www.who.int/reproductivehealth/publications/monitoring/maternal-mortality-2015/en/

[2] Roos N von Xylander S R Why do maternal and newborn deaths continue to occur? Best Pract Res Clin Obstet Gynaecol 2016 36 30 44 10.1016/j.bpobgyn.2016.06.002 27506412

[3] Oyelese Y Ananth C V Postpartum hemorrhage: epidemiology, risk factors, and causes Clin Obstet Gynecol 2010 53 01 147 156 10.1097/GRF.0b013e3181cc406d 20142652

[4] Acosta A A Cabezas E Chaparro J C Present and future of maternal mortality in Latin America Int J Gynaecol Obstet 2000 70 01 125 131 10.1016/S0020-7292(00)00235-6 10884541

[5] UNICEF Maternal mortality data [Internet] 2019[cited 2020 Jul 6]. Available from:https://data.unicef.org/resources/dataset/maternal-mortality-data/

[6] Pino A Albán M Rivas A Rodríguez E Maternal deaths databases analysis: Ecuador 2003–2013 J Public Health Res 2016 5 02 692 10.4081/jphr.2016.692 27747203 PMC5062756

[7] Griggs D Stafford-Smith M Gaffney O Rockström J Ohman M C Shyamsundar P Policy: Sustainable development goals for people and planet Nature 2013 495(7441):305 307 10.1038/495305a 23518546

[8] King M An overall approach to health care for indigenous peoples Pediatr Clin North Am 2009 56 06 1239 1242 10.1016/j.pcl.2009.09.005 19962019

[9] Centro de Estudios de Población y Desarrollo Social Encuesta Demográfica y de Salud Materna e Infantil - ENDEMAIN 2004 [Internet] Quito Cepar 2005[cited 2018 May 10]. Available from:https://cssr-ecuador.org/downloads/2016/11/32.-Encuesta-Demografica-y-de-salud-materna-e-infantil-ENDEMAIN.pdf

[10] Chiriboga S R Incremental health system reform policy: Ecuador's law for the provision of free maternity and child care J Ambul Care Manage 2009 32 02 80 90 10.1097/JAC.0b013e3181994306 19305220

[11] Sanhueza A Roldán J C Ríos-Quituizaca P Acuña M C Espinosa I Social inequalities in maternal mortality among the provinces of Ecuador Rev Panam Salud Publica 2017 41 e97 10.26633/RPSP.2017.97 28614488 PMC6660870

[12] Camacho A V Castro M D Kaufman R Cultural aspects related to the health of Andean women in Latin America: a key issue for progress toward the attainment of the Millennium Development Goals Int J Gynaecol Obstet 2006 94 03 357 363 10.1016/j.ijgo.2006.04.028 16860324

[13] Organización Panamericana de la Salud, Organización Mundial de la Salud Situación de salud: Ecuador 2006 [Internet] 2006[cited 2018 May 10]. Available from:https://www.paho.org/ecu/index.php?option=com_docman&view=download&category_slug=technical-documentation&alias=29-situacion-de-salud-2006&Itemid=599

[14] Oyarce A M Ribotta B Pedrero M Salud materno-infantil de pueblos indígenas y afrodescendientes de América Latina: aportes para una relectura desde el derecho a la integridad cultural Santiago de Chile CEPAL 2010

[15] Castro A Savage V Kaufman H Assessing equitable care for Indigenous and Afrodescendant women in Latin America Rev Panam Salud Publica 2015 38 02 96 109 26581050

[16] Gallegos C A Waters W F Kuhlmann A S Discourse versus practice: are traditional practices and beliefs in pregnancy and childbirth included or excluded in the Ecuadorian health care system? Int Health 2017 9 02 105 111 10.1093/inthealth/ihw053 27993953

[17] Herrera D Hutchins F Gaus D Troya C Intercultural health in Ecuador: an asymmetrical and incomplete project Anthropol Med 2019 26 03 328 344 10.1080/13648470.2018.1507102 30572709

[18] Finerman R D Part 5: pregnancy and childbirth in Saraguro: implications for health care delivery in Southern Ecuador Med Anthropol 1982 6 04 269 278 10.1080/01459740.1982.9987023

[19] Matute S ED Donadi E A Nunes A A Martinez E Z Clinical response to antibiotics in indigenous versus non-indigenous children under 5 years old with community-acquired pneumonia in Otavalo, Ecuador Rev Soc Bras Med Trop 2020 53 e20200038 10.1590/0037-8682-0038-2020 32578709 PMC7310356

[20] Vivar S C Ecuador addresses cultural issues for pregnant women Lancet 2007 370(9595):1302 10.1016/S0140-6736(07)61561-X 17939187

[21] Rosero C MC Quinaluisa S EC Impacto y acogida del parto culturalmente adecuado por el equipo de profesionales de salud del Hospital San Luis de Otavalo en el año 2011–2012 [tesis] Ibarra Universidad Tecnica del Norte 2012[cited 2018 May 10]. Available from:http://repositorio.utn.edu.ec/handle/123456789/2076

[22] Instituto Nacional de Estadística y Censos Estimación de la razón de mortalidad materna en el Ecuador [Internet] Quito INEC 2017[cited 2018 May 10]. Available from:http://www.ecuadorencifras.gob.ec/documentos/web-inec/Poblacion_y_Demografia/Nacimientos_Defunciones/2016/RMM_Nota_metodologica_INEC_2016.pdf

[23] Coast E Jones E Lattof S R Portela A Effectiveness of interventions to provide culturally appropriate maternity care in increasing uptake of skilled maternity care: a systematic review Health Policy Plan 2016 31 10 1479 1491 10.1093/heapol/czw065 27190222 PMC5091340

[24] Gaceta Epidemiológica Ecuador [Internet] Quito Ministerio de Salud Pública. Subsecretaria de Vigilancia de la Salud Pública. Dirección Nacional de Vigilancia Epidemiológica No. 52 2016[cited 2018 May 10]. Available from:https://www.salud.gob.ec/wp-content/uploads/2013/02/GACETA-GENERAL-SE52.pdf

[25] Llamas A Mayhew S The emergence of the vertical birth in Ecuador: an analysis of agenda setting and policy windows for intercultural health Health Policy Plan 2016 31 06 683 690 10.1093/heapol/czv118 26758539 PMC4916315

[26] Waters W F Ehlers J Ortega F Kuhlmann A S Physically demanding labor and health among Indigenous women in the Ecuadorian Highlands J Community Health 2018 43 02 220 226 10.1007/s10900-017-0407-7 28730542

[27] Llamas A Mayhew S “Five hundred years of medicine gone to waste”? Negotiating the implementation of an intercultural health policy in the Ecuadorian Andes BMC Public Health 2018 18 01 686 10.1186/s12889-018-5601-8 29866186 PMC5987654

